# Recent advances in preventing neurodegenerative diseases

**DOI:** 10.12703/r/10-81

**Published:** 2021-12-01

**Authors:** Shih-Ching Chou, Akanksha Aggarwal, Valina L Dawson, Ted M Dawson, Tae-In Kam

**Affiliations:** 1 Neuroregeneration and Stem Cell Programs, Institute for Cell Engineering, 733 North Broadway, Johns Hopkins University School of Medicine, Baltimore, MD 21205, USA; 2 Department of Pharmacology and Molecular Sciences, 725 North Wolfe St., Johns Hopkins University School of Medicine, Baltimore, MD 21205, USA; 3 Department of Biological Chemistry, 725 North Wolfe St., Johns Hopkins University School of Medicine, Baltimore, MD 21205, USA; 4 Department of Neurology, 725 North Wolfe St., Johns Hopkins University School of Medicine, Baltimore, MD 21205, USA; 5 Adrienne Helis Malvin Medical Research Foundation, 228 St. Charles Avenue, New Orleans, LA 70130-2685, USA; 6 Diana Helis Henry Medical Research Foundation, 228 St. Charles Avenue, New Orleans, LA 70130-2685, USA

## Abstract

The worldwide health-care burden of neurodegenerative diseases is on the rise—a crisis created through a combination of increased caseload and lack of effective treatments. The limitations of pharmacotherapy in these disorders have led to an urgent shift toward research and clinical trials for the development of novel compounds, interventions, and methods that target shared features across the spectrum of neurodegenerative diseases. Research targets include neuronal cell death, mitochondrial dysfunction, protein aggregation, and neuroinflammation. In the past few years, there has been a growth in understanding of the pathophysiologic mechanisms of neurodegenerative disorders such as Alzheimer’s disease, Parkinson’s disease, amyotrophic lateral sclerosis, multiple sclerosis, and Huntington’s disease. This increase in knowledge has led to the discovery of numerous novel neuroprotective therapeutic targets. In this context, we reviewed and summarized recent advancements in neuroprotective strategies in neurodegenerative diseases.

## Introduction

Neurodegenerative diseases include a broad range of disorders characterized by neuronal injury or degeneration leading to neurological impairment. These diseases include Alzheimer’s disease (AD), Parkinson’s disease (PD), multiple sclerosis (MS), amyotrophic lateral sclerosis (ALS), and Huntington’s disease (HD) that manifest in millions of people worldwide every year. The common characteristic of these disorders is neuronal loss or impairment resulting in chronic deterioration in memory, locomotor difficulties, psychological impairment, and cognitive defects. Researchers are pursuing a collaborative approach to preserve the function and networks of neural tissues before damage occurs. This neuroprotective approach focuses on the development of strategies that prevent or arrest various types of neuronal cell death mechanisms such as oxidative stress, mitochondrial dysfunction, neuroinflammation, protein aggregation, and defective autophagy, thus limiting disease progression. The purpose of this review is to summarize recent developments in neuroprotective strategies for neurodegenerative diseases.

## Prevention of cell-autonomous neurodegeneration

Cells employ a variety of self-repair mechanisms to maintain and restore physiological homeostasis. When a cell exceeds the ability to overcome stress or damage, intracellular homeostasis collapses and induces a series of cell death signaling cascades. Over the last few years, studies have shown that the apoptotic cell death is not the only pathway dominating neuronal loss in neurodegenerative diseases. Among them, poly (ADP-ribose) (PAR)-dependent cell death, or parthanatos, has been established as being responsible for neuronal loss in a variety of neurological diseases, including AD, PD, ALS, and HD^1^. PARP1 plays a multi-functional role in a variety of cellular processes such as DNA repair pathways, genomic stability, and inflammation^2^. Oxidative stress or nitric oxide production damages DNA, resulting in excessive intracellular PAR accumulation due to PARP1 activation^1^. Several cellular processes result in parthanatos, including PARP1 overactivation, release of apoptosis-inducing factor (AIF) from mitochondria, and co-translocation of AIF and macrophage migration inhibitory factor (MIF) into the nucleus, leading to DNA fragmentation and cell death^2–4^.

Overactivation of PARP1 and PAR accumulation have been observed in the brains of AD patients and mouse models^5,6^, and genetic or pharmacological inhibition of PARP1 protected neurons in AD models^7–9^. Recent studies have also indicated that cells of cognitively impaired patients are more susceptible to H_2_O_2_-induced parthanatos^10^, tissue acidosis-induced amplification of neuronal parthanatos^11^, and robust internal inflammation response following ischemic injuries^12,13^. Moreover, amyloid β (Aβ) causes hippocampal neurotoxicity by inducing oxidative stress–mediated PARP1 activation, which leads to transient receptor potential melastatin-related 2 (*TRPM2*) activation and Ca^2+^ influx and mitochondrial dysfunction^14^. Notably, studies have shown that neurons are protected by PARP1 inhibition, which implies that PARP1 inhibition may have therapeutic value for the treatment of AD.

Recent discoveries in PD models show more direct evidence that parthanatos is the main cell death pathway in pathologic α-synuclein neurodegeneration. In this pathway, PAR is a key mediator, promoting α-synuclein toxicity and fibril transmission, exacerbating neurotoxicity in a feed-forward loop^15^. Interaction between PAR and α-synuclein was also found in post-mortem brains of patients with PD^16–18^. In addition, inhibition of PARP1 may promote α-synuclein autophagy via transcription factor EB-mediated signaling and downregulation of mammalian target of rapamycin (mTOR) signaling, which lessened cytotoxicity of α-synuclein aggregation^17^. Genetic depletion of PARP1 and oral administration of PARP1 inhibitor prevented neurodegeneration and improved motor ability in both sporadic and genetic mouse models of PD^15,17^. Moreover, PAR levels were increased in the cerebral spinal fluid and brains of patients with PD^15^, suggesting that PARP1 could be a theragnostic biomarker and a disease-modifying therapeutic target in PD^19^.

In the ALS brain, expression of PARP1 is increased and localized to a subset of TAR DNA-binding protein 43 (TDP43) inclusions, primary cytological features of ALS^20,21^. Additionally, PAR favors the accumulation and aggregation of hnRNP A1 and TDP43 in stress granules, as observed in patients with ALS^22,23^. Elevated PARP activity is observed in the motor neurons of the ALS spinal cord, and inhibition of PARP mitigates hnRNP A1- or TDP43-mediated neurotoxicity in cell and drosophila models of ALS^22,24^.

In HD, elongated polyglutamine (polyQ) is responsible for huntingtin (htt) protein aggregation and is associated with neuronal inclusions and toxicity^25^. Recently, several co-morbid pathogenic processes were identified in the caudate nucleus of HD brains. The accumulation of damaged DNA, increased PARP1 expression, and localization of htt protein to the DNA damage site were found in conjunction with only weak caspase 3 activation^26^. This finding suggests a pathologic relationship between caspase-independent parthanatos and HD. Moreover, the treatment of PARP1 inhibitor to HD model R6/2 mutant mice showed longer survival and less neuropathologic dysfunction^27,28^. Although these findings suggest a neuroprotective effect of PARP1 in HD, further studies are required to determine the direct relationship of parthanatos to HD.

Dysregulation of PARP1 activation and increased PAR levels contribute to the pathogenesis of various neurodegenerative diseases by promoting protein aggregation and parthanatos. Thus, neuroprotective strategies aimed to inhibit PARP1 activation may have therapeutic potential in those disorders. Many well-characterized PARP inhibitors in clinical use have yet to be tested for use in neurodegenerative disease^29^. These should be considered for neuroprotective treatment for neurological diseases.

## Prevention of non-cell-autonomous neurodegeneration

Glial cells play a central role in neuronal support by maintaining homeostasis, nutrient transportation, and neurogenesis in healthy brains^30^. Importantly, a growing body of research has shown that dysfunctional non-neuronal cells such as microglia and astrocytes directly contribute to neurodegeneration and cell death (so-called non-cell-autonomous neurodegeneration) in a variety of neurodegenerative diseases.

In the brain, microglia are the resident macrophages and primary immune cells. Therefore, they play an important role in neuronal disease. Recent single-cell RNA analysis of central nervous system (CNS) immune cells in AD models discovered a pro-inflammatory signature present in Aβ plaque-associated microglia, also known as disease-associated microglia (DAM), which might play both a toxic and a protective role in AD^31–34^. DAMs have also been observed in other neurodegenerative conditions, including aging, ALS, and frontotemporal dementia (FTD)^33^. Reactive microglia are presented in post-mortem AD brains and have been shown to promote synaptic loss and neuroinflammation in AD^35,36^. Emerging studies suggest that dysregulation of neuroinflammation is modulated by TREM2 and ApoE, which eventually contribute to synaptic loss in multiple AD models^33,37–40^. Additionally, inefficient microglia clearance of pathologic proteins plays an adverse role in the spread of tau in tauopathy and AD^41^. Polarization of microglia toward a neuroprotective M2 type improves neurological deficits in post-ischemic stroke through activation of AMP-activated protein kinase (AMPK) and nuclear factor erythroid 2-related factor 2 (Nrf2) or peroxisome proliferator-activated receptor gamma coactivator 1α (PGC-1α) neuroprotective pathways^42,43^. Many PD-related genes, including *α-synuclein*, *PINK1*, and *parkin*, are expressed in glial cells. Mutated gene products are involved in microglial dysfunction during PD pathogenesis^44^. Also, microglia have been found to modulate the transmission of α-synuclein in the brain^45,46^. Recent spatial transcriptomics of ALS patients and mouse models revealed that changes in microglial gene expression preceded and contributed to motor neuron loss^47^. Accordingly, pharmacological blockage of neuregulin (NRG) receptors present on microglia (associated with ALS disease progression) has been shown to slow disease advancement in an SOD1-ALS mouse model^48^. In summary, microglia are thought to have both beneficial and detrimental functions in neurodegenerative diseases. Thus, induction of DAM or homeostatic microglia signature and subsequent prevention of neurotoxic microglia signature could be promising therapeutic strategies for neuroprotection in neurodegenerative diseases, but the factors associated with heterogenous microglial phenotype will need to be defined in more detail.

Astrocytes are the most abundant population of glial cells in the CNS and perform a broad range of homeostatic functions. Consequently, it is not surprising that the loss of normal astrocyte function is involved in the pathogenesis of neurodegenerative diseases. A 2017 study showed that activated microglia induce the formation of neurotoxic reactive astrocytes by secreting interleukin 1α (IL-1α), tumor necrosis factor α (TNF-α), and C1q^49^. These reactive astrocytes were found in post-mortem brains of human neurodegenerative diseases, including AD, PD, ALS, and HD^49^. The contribution of reactive astrocytes to neurodegeneration has been determined in disease models of PD^50^, AD^51^, ALS^52^, and MS^53^. The presence of reactive astrocytes in numerous disease models creates a prime opportunity for development of neuroprotective therapies that can be shared across multiple neurodegenerative diseases. For example, direct prevention of microglia-mediated naïve astrocyte transformation into reactive astrocytes by Glucagon-like peptide 1 (GLP1) receptor agonist improves behavioral deficits and neurodegeneration in pathologic α-synuclein mouse models of PD^50^. This restorative intervention could be applied to other neurodegenerative disease models. GLP1 receptor agonists are protective in AD mouse models^54^. Additionally, in mouse models for PD, activation of receptor CD44 (expressed on astrocytes) helped reduce nuclear factor kappa B (NFκB) activation and inflammatory response^55^. In another study, dopaminergic neurons produced high levels of prokineticin 2 (PK2) protein^56^. Astrocytes have PK2 receptors; upon ligand-receptor binding, there is a reduction in pro-inflammatory factors and an increase in several antioxidant genes^56^. Genetic depletion of reactive astrocytes markedly extended survival in ALS mouse models^57^. Inhibition of astrocyte reactivity by modulating the JAK2-STAT3 pathway reduced amyloid deposition and synaptic and behavioral deficits in an AD mouse model^58^. In ischemic stroke mouse models, knockout of glutamate-releasing SWELL1 channel present in astrocytes decreased excitotoxicity^59^. More recently, however, it has been identified that the phenotype diversity of astrocytes is observed in brains of neurodegenerative diseases and extends beyond the A1 and A2 phenotypes^60–65^. Thus, further investigation is needed to better understand the molecular mechanisms of reactive astrocytes and their specific role within different neurodegenerative pathologies, especially how neurotoxic signals transduce and are shared across multiple neurodegenerative conditions.

## Conclusions

**Figure 1.**
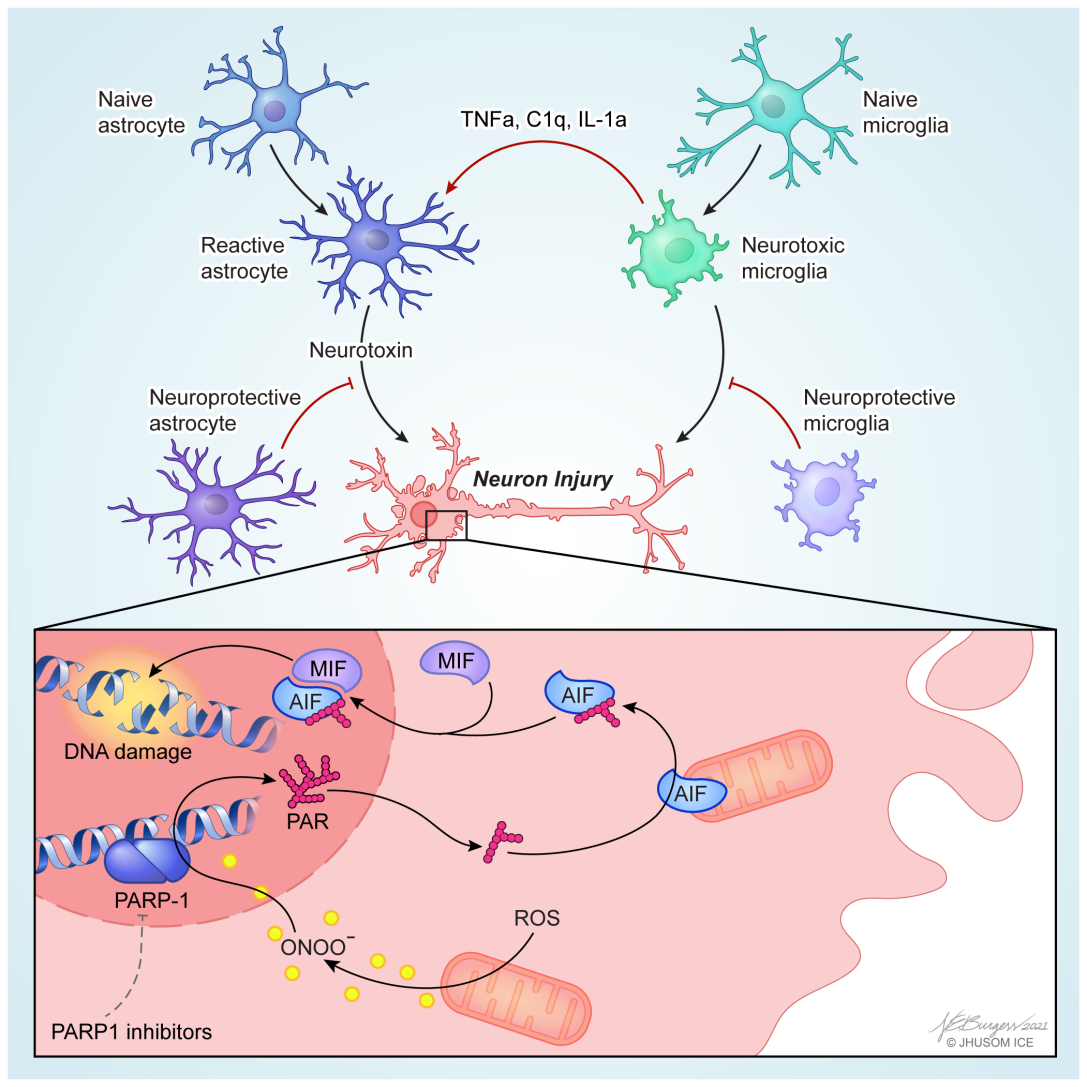
Cell-autonomous and non-cell-autonomous neurodegeneration. PARP1-dependent cell-autonomous mechanisms of neurodegeneration (bottom). Neuron injury stressors such as an oxidative stress or aggregated proteins activate nitric oxide synthase that produces nitric oxide and then peroxynitrite (ONOO^−^), resulting in overactivation of PARP1. Accumulated poly (ADP-ribose) (PAR) polymers synthesized by overactivated PARP1 translocate from the nucleus to the cytoplasm and mitochondria, where it binds to and induces mitochondrial release of apoptosis-inducing factor (AIF). AIF-bound macrophage migration-inducing factor (MIF) nuclease translocates into the nucleus, where MIF cleaves genomic DNA into large-scale fragments, causing cell death. Inhibition of PARP1 can protect neurons in a variety of neurodegenerative diseases (see ‘Prevention of cell-autonomous neurodegeneration’ section). Non-cell-autonomous mechanisms of neurodegeneration mediated by microglia or astrocytes (top). Induction of disease-associated microglia or homeostatic microglia and subsequent prevention of neurotoxic microglia could be promising neuroprotection strategies in neurodegenerative diseases. Alternatively, activated microglia induces the formation of neurotoxic reactive astrocytes by secreting interleukin 1α (IL-1α), tumor necrosis factor α (TNF-α), and C1q. Reactive astrocyte-targeted neuroprotection could be achieved by microglial inhibition of formation of neurotoxic reactive astrocytes and induction of neuroprotective astrocytes. PARP, poly (ADP-ribose) polymerase; ROS, reactive oxygen species.

Neurodegenerative diseases are the result of a number of factors, including genetic mutations, neuronal cell death, mitochondrial dysfunction, protein aggregation, flawed protein recycling, and innate immune responses due to activation in glial cells. Thus, neuroprotection from cell-autonomous neurodegeneration could be achieved by directly targeting degenerating neurons and from non-cell-autonomous neurodegeneration by targeting their neighboring glial cells (Figure 1). Thus, a multifaceted approach targeting both cell-autonomous and non-cell-autonomous mechanisms may be required to prevent or slow neurodegeneration. During the previous decade, consistent and focused studies have revealed the causal factors in neurodegenerative diseases. Understanding the molecular mechanisms of neurodegeneration is an essential step forward in the development of novel neuroprotective therapies. The last couple of years have seen advancements in both research and clinical understanding of potential novel neuroprotective therapeutics. Owing to tremendous effort, these therapies have progressed beyond the lab and into the clinical testing stage. Despite this progress, therapies to prevent or decrease disease progression and restore neuronal function remain a challenge and an ongoing focus in both research and clinical practice. Thus, further investigation into the neurodegenerative pathways and the identification and development of neuroprotective agents are needed to develop promising disease-modifying therapeutic approaches for the treatment of neurodegenerative disease.
